# Evaluating the likelihood of pediatric sacral nerve stimulator explantations due to cure or complications: a survival analysis of 13-year institutional cohort

**DOI:** 10.1007/s00345-025-05916-7

**Published:** 2025-08-28

**Authors:** Jin Kyu Kim, Konrad M. Szymanski, Rosalia Misseri, Shelly J. King, Nikhil V. Batra, Martin Kaefer, Mark P. Cain, Richard C. Rink, Joshua Roth, Pankaj Dangle, Kirstan Meldrum, Benjamin M. Whittam

**Affiliations:** Department of Urology, Riley Hospital for Children, 702 Barnhill Drive, Indianapolis, IN 46202 USA

**Keywords:** Sacral neuromodulation, SNM, Bladder dysfunction, Explantation

## Abstract

**Introduction:**

Sacral neuromodulation (SNM) is a treatment option for children with refractory bladder and bowel dysfunction. Prior investigations have shown children may achieve cure of their symptoms following SNM implants and subsequently have their devices explanted. Herein, we present a 13-year experience of pediatric SNM placements and evaluate the likelihood of SNM explantation for any cause, for symptom resolution or complications.

**Methods:**

An institutional retrospective review of children who underwent a 2nd stage SNM placement between November 2012 and January 2025 was performed. Reasons for SNM explantation was categorized as a cure or complication. Competing-risk time-to-event analysis was used.

**Results:**

There were 129 SNM placements at a median of 10 years old (IQR 8.1–12.7); 88 were females (68.2%) and 41 required SNM revision (31.8%). Median follow-up was 3.5 (IQR 2.0–5.3) years. Subsequently, 46 underwent SNM explantation (35.7%). On survival analysis, median time to explantation (50%) was 6.0 (IQR 4.6–7.3) years. Among explanted, 34 were due to symptom resolution (73.9%) and 13 due to complications (4 infections; 4 pain at site; 3 for MRI requirements; 1 clinically ineffective). On competing risks analysis, 72.5% of the explantations at 6 years were for cure and 27.5% for complications. The 6-year explantation risk was 36.3% for cure and 13.8% for complications. Among 17 children who provided data after device explanation following cure (response rate: 51.5%), 16 (94%) had sustained symptom resolution at a median of 3.8 years (IQR 1.3–5.3) after explantation.

**Conclusion:**

Approximately quarter of children with SNM placement achieved cure with increasing probability with follow-up time. More than 70% of explantations are due to cure and less than 10% were due to infections. There is high likelihood of sustained symptom resolution following explantation for cure. SNM remains a safe and viable option for children with refractory BBD with potential for cure.

## Introduction

Pediatric bladder bowel dysfunction (BBD) encompasses a broad spectrum of lower urinary tract symptoms, including urinary incontinence, urgency, frequency, and retention, which significantly impact a child’s physical health, emotional well-being, and overall quality of life. Left untreated, BBD may contribute to complications such as recurrent urinary tract infections, vesicoureteral reflux, renal damage, and social stigma. Management strategies for BBD often require a combination of behavioral interventions, pelvic floor physical therapy, pharmacologic treatments, and in severe cases, catheterization or reconstructive surgery. Despite these approaches, a small but signficant subset of pediatric patients remains refractory to conventional therapies, necessitating alternative interventions [1].

Sacral nerve modulation (SNM) has emerged as an effective neuromodulatory therapy targeting BBD by altering afferent and efferent pathways involved in urinary control. SNM involves delivering mild electrical impulses to the sacral nerves to modulate bladder activity, thus restoring functional voiding patterns. While extensively studied in adult populations, pediatric applications have gained traction over the past decade, with studies demonstrating favorable outcomes in symptom relief, continence rates, and quality of life improvements. Approximately 70–80% of pediatric SNM recipients experience significant clinical improvement, with complete symptom resolution (‘cure’) rates documented in up to 40% of cases [1–4]. However, these studies primarily focus on short- to mid-term outcomes, leaving gaps in the literature regarding long-term device retention, explantation trends, especially for cure, and the durability of symptom resolution post-explantation [5].

Given the increasing adoption of SNM in pediatric populations, our study aims to provide an extended follow-up analysis of children who underwent SNM therapy for refractory bladder dysfunction who subsequently underwent SNM explantation. We hypothesize that with increasing follow-up, more patients will have their devices explanted for cure.

## Methods

Following approval from institutional research ethics board (IRB#1,605,024,102), a retrospective review of children (< 18 years of age) who underwent a 2nd stage SNM placement between November 2012 and January 2025 was performed.

Our institutional protocol for SNM required that patients had refractory BBD (defined as no significant improvement following behavioral or dietary modifications, biofeedback/pelvic physiotherapy, aggressive constipation management, and pharmacotherapy with anticholinergics for ≥ 2 years). In all patients, spinal MRI was performed to rule out occult spina dysraphism and if there were any signs suggestive of tethered cord, neurosurgery referral was initiated. However, if symptoms persisted following neurosurgical intervention, SNM was offered after informed consent was obtained thorough counseling regarding its off-label use in pediatric population.

All patients underwent a trial stage (1st stage) procedure to observe for symptom improvement over a 2-week period. Those with less than 50% improvement of symptoms did not receive a permanent SNM device implant and were excluded from final analysis.

Briefly, the operative technique for 1st stage procedure involves lead placement adjacent to the S3 nerve root under general anesthesia and confirmation of bellows response and plantar flexion of the great toe with stimulation of ipsilateral side. If > 50% improvement is noted in BBD symptoms with an external generator for 2 weeks, a 2nd stage procedure is offered and an internal generator was placed in the subcutaneous space in the contralateral superior gluteal fold.

Following implantation, close follow-up is ensued and for those with complete symptom resolution after 1 year of implantation, the device was turned off and for those with sustained symptom resolution for ≥ 6 months (“cure”) were we offered device explantation.

Reasons for SNM explantation was categorized as a cure or complication. Kaplan–Meier survival curve was created to evaluate time to explantation for any reasons. Competing-risk time-to-event analysis was used to compare explantation due to cure or complication. Pharmacotherapy (surrogate measure of symptom resolution/improvement) at last follow up was evaluated based on explantation status using Fisher’s Exact Test.

All analyses were performed using R Studio (version 2024.04.0 + 735), with an alpha value of 0.05.

## Results

A total of 139 patients underwent initial first stage procedure. Ten patients did not undergo second stage procedure and were excluded. There were 129 SNM placements at a median of 10.0 years old (IQR 8.1–12.7); 88 were females (68.2%) and 41 required SNM revision (31.8%). Median follow-up was 3.5 (IQR 2.0–5.3) years.

Subsequently, 47 underwent SNM explantation (36.4%). Among those who did and did not have their device explanted, there was no difference in these characteristics (age, gender, filum section, time from filum section to reimplant, revision, follow-up period), including previous sectioning of the filum terminale (Table 1).

**Table 1:** Summary of clinical characteristics between those with devices remaining and devices explanted

Clinical Characteristics	Device Remaining (N = 82)		Device Explanted (N = 47)		p
	N, Median	%, IQR	N, Median	%, IQR	
Age at Implantation (Years)	8.0	7.2–10.7	9.8	8.4–12.6	0.192
Gender (Female)	27	32.9%	14	29.8%	0.846
Filum section	17	20.7%	7	14.8%	0.492
Time from filum section to implant (years)	2.4	2.1–5.6	0.9	0.6–2.1	0.172
Revision	31	37.8%	10	21.3%	0.078
Follow up (Years)	3.5	1.3–5.9	3.6	2.6–5.2	0.372

**Fig. 1:**
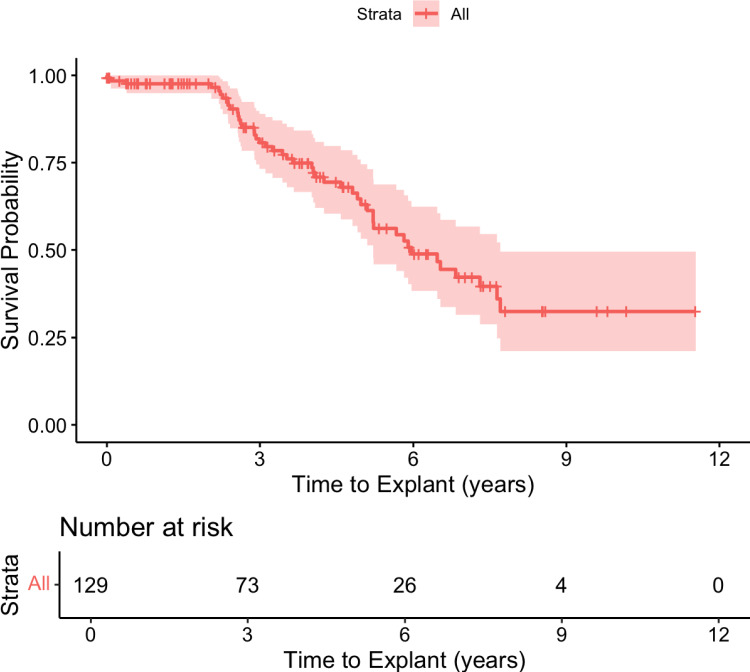
Kaplan–Meier survival curve analysis of time to device explantation

On Kaplan–Meier survival curve analysis, median time to explantation (time of 50% survival) was 6.0 (IQR 4.6–7.3) years. Among explanted, 34 were due to symptom resolution (72.3%) and 13 due to complications (27.7%; 4 infections; 4 pain at site; 3 for MRI requirements; 1 clinically ineffective; Fig. 1).

**Fig. 2:**
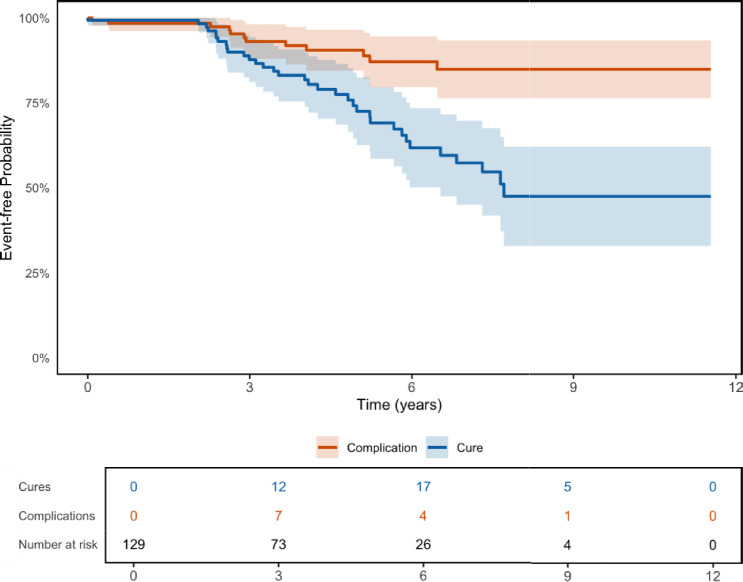
Competing risks analysis of explantation for symptom resolution (cure) and complication (Gray’s test p = 0.001)

On competing risks analysis, 72.5% of the explantations at 6 years were for cure and 27.5% for complications. The 6-year explantation risk was 36.3% for cure and 13.8% for complications (Fig. 2). There was higher likelihood of having device explantation for cure (Gray’s test p = 0.001). Majority of explantations related complications (58%) occurred within the first 3 years.

Among the 34 patients who achieved cure and had their devices explanted at a median of 3.2 years (IQR 1.4–5.2), 18 responded to post-explantation follow-up phone call evaluation (response rate: 52.9%) at median of 3.8 years (IQR 1.3–5.3) following explantation. Among those who responded, 94.4% (17/18) had sustained symptom resolution and did not take any medications for BBD. One patient described recurrence of milder symptoms and was taking mirabegron for urgency. Among non-respondents, 5/16 (31%) had follow up with a healthcare provider within our state, with median time of 1.2 years (IQR 0.66–4.32) since explantation. One of these patients continued to take oxybutynin for nocturnal enuresis. The other four patients were documented to have no genitourinary symptoms. Overall, among 23 patients who had any follow up post explantation at median of 4.3 years (IQR 0.66–5.33), two patients (9%) had symptoms that were well managed on medication monotherapy while remainder (91%) had sustained symptom resolution.

As a surrogate measure of symptom resolution/improvement, pharmacotherapy at last follow-up was evaluated among patients (Table 2). Compared to those without device explantation (20.7%), those who had device explantation for complications had a higher proportion of patients on pharmacotherapy (46.2%) and those with device explantation for cure had a lower proportion of patients on pharmacotherapy (2.9%; p = 0.001).

**Table 2:** Comparison of proportion of patients on pharmacotherapy based on explantation status at last follow up (p = 0.001)

	Explanted for Cure (n = 34),		Explanted for Complications (n = 13)		No Explantation (n = 82)	
	N	%	N	%	N	%
Pharmacotherapy at last follow up	1	2.9%	6	46.2%	17	20.7%
Desmopressin	1	2.9%	0	0.0%	6	7.3%
Anticholinergic	0	0.0%	4	30.8%	8	9.8%
Beta-3 Agonist	0	0.0%	1	7.7%	2	2.4%
Tamsulosin	0	0.0%	1	7.7%	3	3.7%

## Discussion

Our study confirms that SNM is associated with long-term therapeutic benefits in pediatric patients with bladder dysfunction. Previously, device explantation rate for cure was reported to be more common than explantation for complication-related such as infection or pain [4]. With increasing follow up, there appeared to be higher likelihood of device removal for cure [5]. Our study corroborates this finding and confirm our hypothesis that cure rates steadily increase with time, while majority of explantation for complications occur in within the first 3 years.

Moreover, while our study corroborates existing data on SNM effectiveness, it also provides important insights into device retention and complications. The 31.8% revision rate and 35.7% explantation rate are consistent with previously reported hardware-related complications in pediatric cohorts [3, 4]. It should be noted that while explantation for complications occurred in a minority of our patients, 23% (3/13) of these patients underwent explantation due to need for MRI. As newer versions of SNM devices are MRI compatible, it is likely that this complication will no longer be relevant in future populations. [6]

Furthermore, our long-term post-explantation data indicate that SNM-induced symptom resolution remains durable; when children experienced cure and had their SNM subsequently explanted at approximately 4 years after implantation, more than 90% with follow up maintaining resolution of symptoms after explantation. This finding underscores SNM’s potential not only as a symptomatic therapy but as a long-term modulator of bladder function. This also suggests that device explantation for cure should be regarded as safe as results are sustainable for more than 3 years in children with devices explanted in our cohort. Future research should explore predictors of long-term success, refine patient selection criteria, and investigate strategies to minimize device-related complications while enhancing therapy persistence. Our study was underpowered for this analysis.

In our earlier investigations, we found that while there was no statistically significant difference, more patients with filum sections had kept their device [5]. However, with a larger cohort, we can see more clearly that there is no difference in likelihood of explantation based on history of filum sectioning. While there is mixed observational evidence and negative randomized trial evidence on the effects of filum sectioning on bladder dysfunction and urinary symptoms, there is heterogeneity in practice patterns amongst neurosurgeons which make it difficult to capture the true effect of filum sectioning in this cohort [7–10]. However, as every patient in this study had ongoing bladder dysfunction refractory to all prior treatments, including filum sectioning, our data suggests that SNM remains a helpful tool regardless of a history of filum sectioning.

This study has several limitations. As a retrospective study, it is subject to sampling bias; however, we ensured to minimize this by evaluating all patients who underwent device implantation within our defined time-period. The sample size is small, with 129 patients; however, considering the paucity of pediatric SNM literature available, this would still represent a significant number of pediatric SNM experience. Due to the nature of the study, with the primary goal being device explantation, there was no valid comparator group. Our institution currently does not offer potential therapies with comparative effects such as peripheral tibial nerve stimulation, which may serve as a comparator therapy group. Another significant limitation of our study is the method of post-explantation follow-up. A validated survey approach was not feasible due to the challenge of directly reaching pediatric patients, as most contact information was linked to parents. As a result, most symptom resolution and long-term success outcomes were based on parental testimony rather than direct patient-reported outcomes. This introduces the possibility of recall bias or underreporting of recurrent symptoms. This remains a significant limitation; as the study was retrospective in nature and aimed to evaluate the clinical experience of pediatric SNM, we did not plan for a standardized pre- and post-operative validated assessment tools. Future studies should consider implementing structured, validated assessment tools to obtain more precise and objective measures of post-explantation outcomes.

## Conclusion

Approximately quarter of children with SNM placement achieved cure with increasing probability with follow-up time. Minority of explantations were for complications, with less than 10% infection risk. There is high likelihood of sustained symptom resolution following device explantation for cure. SNM remains a safe and viable option for children with refractory bladder or bowel dysfunction.

## Data Availability

Data will be made available upon reasonable request to the corresponding author and establishment of data sharing agreement between institutions.
